# The Brown Bear and Hibernating Mammals as a Translational Model for Human Resilience: Insights for Space Medicine, Critical Care, and Austere Environments

**DOI:** 10.3390/biology14101434

**Published:** 2025-10-17

**Authors:** Jainam Shah, Ryung Lee, Sachin Pathuri, Jason Zheng, Joshua Ong, Alex Suh, Kimia Rezaei, Gagandeep Mudhar, Andrew D. Parsons, Jaewoo Park, Andrew G. Lee

**Affiliations:** 1 Albert Einstein College of Medicine, Bronx, NY 10461, USA; gagandeep.mudhar@einsteinmed.edu (G.M.); andrew.parsons@einsteinmed.edu (A.D.P.); jaewoo.park@einsteinmed.edu (J.P.); 2 Department of Medicine, Jacob’s School of Medicine and Biomedical Sciences, Buffalo, NY 14203, USA; 3 Creighton University School of Medicine, Phoenix Regional Campus, Phoenix, AZ 85012, USA; sachinpathuri@creighton.edu; 4 California University of Science and Medicine, Colton, CA 92324, USA; jason.zheng@md.cusm.edu; 5 Department of Ophthalmology and Visual Sciences, University of Michigan Kellogg Eye Center, Ann Arbor, MI 48105, USA; ongjo@med.umich.edu; 6 Department of Ophthalmology, USC Roski Eye Institute, Keck School of Medicine, University of Southern California, Los Angeles, CA 90333, USA; alexander.suh@med.usc.edu; 7 University of California Riverside School of Medicine, Riverside, CA 92507, USA; kimia.rezaei@medsch.ucr.edu; 8 Department of Ophthalmology, Blanton Eye Institute, Houston Methodist Hospital, Houston, TX 77030, USA; aglee@houstonmethodist.org; 9 Department of Ophthalmology, Baylor College of Medicine and the Center for Space Medicine, Houston, TX 77030, USA; 10 The Houston Methodist Research Institute, Houston Methodist Hospital, Houston, TX 77030, USA; 11 Departments of Ophthalmology, Neurology, and Neurosurgery, Weill Cornell Medicine, New York, NY 10065, USA; 12 Department of Ophthalmology, University of Texas Medical Branch, Galveston, TX 77555, USA; 13 University of Texas MD Anderson Cancer Center, Houston, TX 77030, USA; 14 Texas A&M College of Medicine, Bryan, TX 77807, USA; 15 Department of Ophthalmology, The University of Iowa Hospitals and Clinics, Iowa City, IA 52242, USA; 16 Department of Ophthalmology, University of Buffalo, Buffalo, NY 14214, USA

**Keywords:** hibernation, brown bear, *Ursus arctos*, space medicine, disuse resilience, neuro-ocular protection, metabolic suppression, RBM3, synthetic torpor, translational physiology

## Abstract

**Simple Summary:**

Spaceflight, bed rest, and critical illness each cause the body to be severely stressed. Muscle atrophy, bone loss of density, weakening of the heart, failure of the immune system, and sight impairment are very common. Diet and exercise interventions can halt these occurrences, but cannot hold them off entirely. Hibernating animals, however, like brown bears, tolerate months of immobility, fasting, and very low heart rates with no long-term consequences. They emerge from hibernation with preserved muscle strength, unbroken bones, and functioning brain and eyes. Even the waste products are recycled into useful nutrients rather than being lost. In this review, we explain how hibernating bears and smaller mammals achieve such resilience during hibernation. We highlight the natural mechanisms that keep their organs healthy and explore how these lessons may translate into novel clinical approaches. Applications range from protecting astronauts on extended spaceflight to helping recovery in critically ill patients and avoiding common age-related conditions such as osteoporosis, muscle wasting, and loss of memory. Studies of hibernators allow medicine to move beyond mending after damage and into preventing damage, and these consequences extend to both human spaceflight and terrestrial health.

**Abstract:**

Long-term spaceflight induces multisystem stress, including cardiovascular deconditioning, skeletal muscle atrophy, immune suppression, and neuro-ocular syndromes. Current countermeasures reduce symptoms but cannot replicate the synergistic resilience needed for extended missions or critical illness. Hibernating animals, specifically brown bears (*Ursus arctos*), survive prolonged immobility, starvation, and bradycardia without resultant pathology. This review incorporates adaptations observed in bears and certain torpid species, including reversible insulin resistance, suppression of muscle atrophy genes MuRF1 and Atrogin-1, and maintenance of the heart despite seasonal production decline. The thirteen-lined ground squirrels (*Ictidomys tridecemlineatus*) maintain retinal structure and synaptic stability throughout torpor, avoiding neuro-ocular complications despite prolonged inactivity. Mechanisms span from RBM3-dependent synaptic maintenance, titin isoform remodeling under the control of RBM20, mTOR and FOXO pathway regulation, remodeled hydrogen sulfide metabolism, and microbiome-mediated nitrogen salvage. These adaptations are different from human adaptation to microgravity and disuse and offer translational candidates for synthetic torpor, probiotic engineering, neuroprotection, and protein-sparing therapy. Hibernators are not passive stress subjects; they perform coordinated anticipatory responses in multiple organs. Comparing these systems in large and small hibernators, we aim to uncover a biologically realistic path to human resilience. These findings guide a shift from reactive, pharmacological measures for preserving human health during space flight, intensive care, and extreme environments towards proactive, biologically initiated measures.

## 1. Introduction

Human spaceflight introduces a cascade of physiological stresses that affect nearly every organ system. Microgravity disrupts fluid distribution, reduces mechanical loading, and shifts circadian rhythms, while chronic exposure to radiation and confinement accelerates systemic deterioration [1]. Cardiovascular adaptations include central venous congestion, reduced stroke volume, and orthostatic intolerance after return to Earth [2]. Skeletal unloading triggers rapid bone demineralization and muscle atrophy, which are incompletely reversed by exercise and pharmacologic countermeasures [3]. The nervous system is similarly affected, with visual impairment and intracranial pressure change included under the spectrum of spaceflight-associated neuro-ocular syndrome [1,4]. Immune dysregulation is also increasingly evident, with astronauts showing impaired lymphocyte signaling, altered cytokine balance, and stress-related immune aging [5,6]. These changes highlight the systemic vulnerability of the human body to prolonged microgravity exposure. Radiation also adds to these risks by accelerating vascular aging, allowing DNA damage, and reducing CNS repair capacity, making it one of the most critical long-term barriers to interplanetary travel [7]. Beyond spaceflight, many of these physiologic challenges also occur in terrestrial scenarios, such as critical illness, prolonged ICU stay, and austere or resource-limited environments, in which humans undergo metabolic stress, immobilization, or confinement.

Animal models have been invaluable to the study of these mechanisms. Rodent hindlimb unloading has provided decades of musculoskeletal and vascular deconditioning data, but limited life spans and ecological fidelity restrict translational potential [8]. Larger animal analogs, such as swine or non-human primates, model either cardiovascular or musculoskeletal stress but remain costly and ethically limited. The need for integrative comparative models that portray resilience rather than susceptibility has therefore become increasingly evident [2,9,10]. However, these methods isolate individual organ responses, while human spaceflight at the systems level challenges physiology, and models are needed that can show coordinated robustness between organ systems [2].

**Figure 1:**
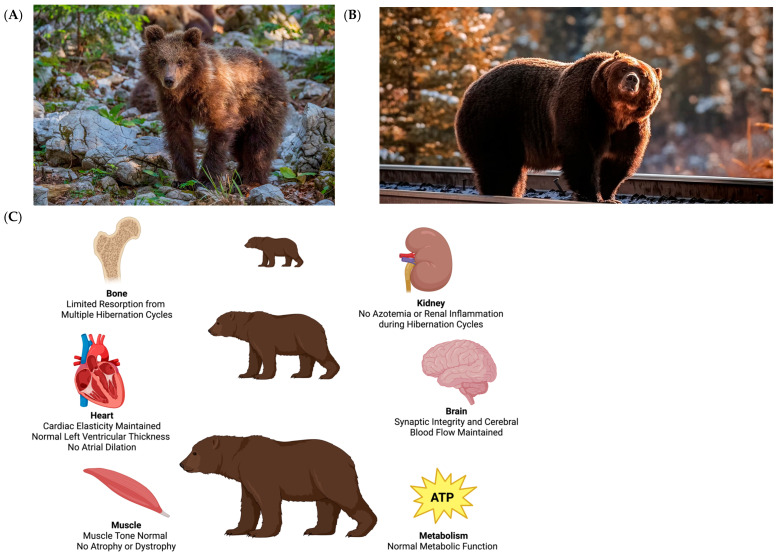
Developmental and resilience of the brown bear (*Ursus arctos*). (**A**) Brown bear cub in a forest environment. (**B**) Fully grown adult male brown bear, colloquially known as “The Boss,” is one of the largest free-ranging individuals in the Canadian Rockies. These images illustrate the striking growth of the species, which undergoes hibernation from cubhood to adulthood. (**C**) Schematic representation of the bear life cycle across successive hibernations, showing the major physiologic stressors (immobility, fasting, bradycardia, anuria) and the preservation of key organ systems (muscle, bone, heart, kidney) across development. Image (**A**) used under Creative Commons Attribution 4.0 International License (https://creativecommons.org/licenses/by/4.0/deed.en, accessed on 27 August 2025) from Sharp Photography (sharpphotography.co.uk, accessed on 27 August 2025); image (**B**) used with permission from (https://freerangeamerican.us/, accessed on 27 August 2025) and (https://banffnationalpark.com/, accessed on 27 August 2025). Panel (**C**) created in BioRender. Shah, J. (2025) https://BioRender.com/kg5c1s4.

Brown bears (*Ursus arctos*) represent one of the most compelling models for resilience, surviving prolonged immobility and fasting from cubhood to adulthood without lasting organ dysfunction, as shown in Figure 1. During hibernation, they experience months of near-total inactivity, fasting, and bradycardia, but they emerge without the systemic deconditioning that affects humans under similar conditions [11]. Brown bears are large-bodied omnivores primarily found across Eurasia and North America, typically weighing 100–300 kg, with some subspecies reaching over 500 kg [12]. They hibernate for up to 5–7 months every winter, utilizing stored fat for energy and undergoing a state of metabolic suppression without full torpor. Compared to smaller hibernators, brown bears maintain a relatively high core body temperature (~33–34 °C) and remain partially alert, exhibiting shallow, reversible physiologic adaptations [13]. These traits make them uniquely suited for modeling human-relevant disuse conditions. Many of the hibernation strategies described are also present in other species discussed in this review, such as the American black bear (*Ursus americanus*), Arctic ground squirrel (*Urocitellus parryii*)*,* thirteen-lined ground squirrel (*Ictidomys tridecemlineatus*), and Djungarian hamster (*Phodopus sungorus*), each contributing smaller, albeit unique insights into organ-specific or molecular adaptations.

Echocardiographic and hemodynamic studies of free-ranging Scandinavian brown bears show preserved myocardial structure despite seasonal heart rates falling below 30 beats per minute and cardiac output declining to approximately one-quarter of active-season levels [14,15]. Unlike humans with disuse cardiomyopathy, bears do not experience atrophy and recover normal function within days of arousal [9]. Their immune system also exhibits resilience, managing inflammatory signaling without succumbing to chronic dysfunction, a phenomenon linked to stress-resistance pathways and potentially protective against aging-like immune decline [15,16]. Seasonal modulation of circadian and metabolic rhythms further illustrates a systemic reprogramming that synchronizes cardiovascular, musculoskeletal, and immune maintenance during winter dormancy [15,17]. Even when ecological or human-induced stresses alter denning behavior, bears display flexible physiological responses that preserve energy balance and organ integrity [18].

These characteristics extend beyond hibernation itself. Bears exhibit coordinated adaptations across cardiovascular, metabolic, and immune systems that facilitate survival during intervals of prolonged immobility and caloric deprivation [9]. This resilience is in direct contrast with the rapid decline seen in astronauts, rendering bears a compelling comparative model for the discovery of protective mechanisms. Notably, being able to experience reversible multi-system suppression without residual pathology offers a biological template for designing countermeasures. While brown bears are the central species discussed in this review, we also incorporate findings from other hibernators where they offer superior mechanistic detail or complementary insight, particularly in neuroprotection, renal metabolism, and circadian regulation.

The objective of this review is threefold: first, to recapitulate the current understanding of cardiovascular, musculoskeletal, ocular, immune, metabolic, and circadian risks to humans during spaceflight; second, to outline the physiological strategies employed by brown bears and select other species that preserve multi-system integrity during prolonged disuse and metabolic suppression; and third, to highlight translational opportunities where bear and hibernator physiology can guide countermeasures to sustain astronaut health in spaceflight and, where relevant, inform terrestrial contexts such as aging, immobilization, and chronic disease [2,15,17].

## 2. Bear Hibernation: A Physiologic Blueprint for Disuse Resilience

### 2.1. The Problem: Multi-System Decline in Disuse States

When humans stay in microgravity for extended periods, have bedrest within a confined environment, or experience immobilization within settings, such as the intensive care unit (ICU), the human body begins breaking down in a manner that affects nearly every organ system negatively. This also holds true during spaceflight, which places its own stresses: fluids move to the head, muscles and bones are unloaded, sleep–wake cycles are disturbed, and radiation continues to cause cell damage [2,4]. These insults do not necessarily take years to become evident, as measurable effects have been noted in days [19].

Compromise of the cardiovascular system is typically the first consequence. Plasma volume decreases, baroreceptor gain is decreased, and stroke volume is decreased, making many astronauts vulnerable to lightheadedness or syncope upon return to Earth [20]. Highly controlled bedrest experiments reproduce the same orthostatic intolerance, highlighting the model’s fidelity and reliability [21]. Simultaneously, bone and muscle both quickly atrophy. Astronauts lose 1–2 percent of weight-bearing bone density each month, and skeletal muscle fibers shrink in size even with exercise countermeasures [3,22]. ICU-immobilized patients endure the same effects, with catabolic breakdown inducing prolonged weakness months following discharge [23].

The nervous system is also vulnerable. A small percentage of astronauts exhibit visual and intracranial changes under the umbrella of the term “spaceflight-associated neuro-ocular syndrome” (SANS) [24]. Imaging and diagnostic evaluation in SANS has revealed unilateral and bilateral optic disc swelling, posterior globe flattening, choroidal and retinal folds, hyperopic refractive error shifts, and areas of ischemic retina [24]. Head-down tilt maneuvers reproduce these alterations, thus showing that cephalad fluid shifts alone can reorganize neural tissues [19,25]. Immunity also declines, with evidence of dysregulated lymphocyte signaling and altered cytokine balance that increase infection risk and mimic premature immune aging [5]. These patterns have similarly occurred in long-term bedrest and confinement studies [26].

To study these problems, rodent hindlimb unloading has been the foremost ground-based analogue. It has clarified numerous pathways of muscle atrophy, bone loss, and vascular change [8]. Large animal systems like swine and primates have yielded more data but remain costly and of limited access [27]. The ongoing drawback of all these systems is that they are simulating human fragility rather than resistance. This limitation has shifted research interest to hibernating mammals, specifically brown bears, that make it through months of inactivity without the same extreme systemic deterioration [9,14,15,16,17,28].

### 2.2. The Bear’s Strategy: Coordinated Multi-System Preservation

**Figure 2:**
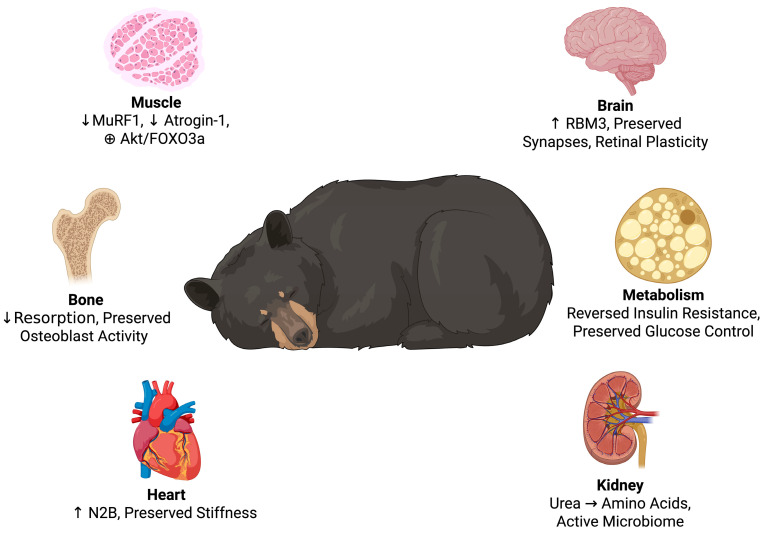
Multisystem adaptations in hibernating bears. The key physiological adaptations of hibernation are inhibition of muscle breakdown, ongoing bone remodeling, cardiac stiffness provided by titin isoform switches, nitrogen recycling by the kidneys, reversible insulin resistance with preserved glucose regulation, and neuroprotection via RBM3-mediated synaptic and retinal plasticity. Created in BioRender. Shah, J. (2025) https://BioRender.com/mzohwp8.

During hibernation, brown bears can spend months in physical inactivity, hibernation fasting, and anuria, but emerge unharmed with conserved muscle, stable metabolism, and without any sign of organ failure [16,29]. It is not a coincidence that this is possible; Figure 2 illustrates the multisystem adaptations that allows it to occur. Beneath the surface lies a tightly regulated and seasonal program, one that oversees alterations via the heart, muscle, kidney, endocrine system, and others [28]. Core body temperature only drops slightly, from ~37 °C to 33–34 °C, slowing metabolic rate by as much as 75% without triggering the deeper torpor or enzyme inactivation seen in the smaller hibernators [30,31]. Moreover, brain activity remains detectable, with EEGs showing low-frequency activity and maintained arousability [32]. In essence, the bear is not asleep; it’s merely slowed down.

This moderate metabolic suppression also extends to the cardiovascular system. Heart rate slows dramatically to 8–14 beats per minute without any change in cardiac architecture [14,33]. This occurs because bears switch to the N2B isoform of titin, a stiffer version of the giant protein that maintains ventricular elasticity and prevents dilation on prolonged bradycardia [33].

Muscle bulk, which is rapidly lost in hospitalized humans, is surprisingly well-preserved. There is no significant atrophy during the denning time, owing to a complex gene expression pattern that suppresses proteolysis and enhances oxidative metabolism [11,34]. The muscle enzymes in the skeletal muscle fluctuate seasonally, as glycolysis and lipid pathways are remodeled to match the bear’s energetic needs [35]. In spite of remaining totally motionless, bears still exhibit periodic, low-level muscle activity that may be involved in such preservation [28].

These adjustments also extend to the endocrine system. Hibernating bears have a reversible insulin resistance: leptin and insulin concentrations fall, but blood glucose remains in the normal range [35,36]. This adjustment fully reverses after arousal, and there is no lingering metabolic impairment [37]. Paradoxically, levels of SHBG, which regulate hormone availability, rise during hibernation, which could help buffer sex hormone action during low-energy states [37].

Remarkably, the kidneys stop producing urine for months, but bears avoid the effects this would have in humans. They do not accumulate nitrogenous waste or suffer electrolyte disturbance. Instead, urea is salvaged by intestinal bacteria into amino acids that are reabsorbed, a lovely mechanism of nitrogen economy [38,39,40]. Blood urea nitrogen and creatinine concentrations are maintained within protective levels, and renal function is regained after hibernation with not a trace of damage or long-term effect [40].

Lipid regulation is also maximized for protective effects. While hibernating bears retain more cholesterol, they avoid vascular damage. This can be explained by increased antioxidant action and modified lipoprotein metabolism that collectively suppress atherosclerosis [41]. Moreover, these changes are not isolated. A transcriptomics dataset in 13 tissues of free-ranging hibernating bears illustrates a unique coordination pattern, as the kidney, muscle, adipose tissue, and liver all undergo coordinated changes to support less metabolic burden, maintain proteome homeostasis, and prevent inflammation [42]. Not only do multiple organs adjust, but they also adjust in combination synergistically, making this a highly intriguing mechanism to study.

The bear not only slows down, but it enters a stable and defensive mode that is maintained through body systems. When conditions improve and supplies are abundant, it leaves that state with ease. Humans, however, will lose considerable muscle and cardiovascular capacity after a couple of weeks of bedrest. The bear sleeps in suspended animation for six months and lumbers out prepared to go without the identical severe systemic debilitation [9,14,15,16,17,28].

### 2.3. Translational Opportunities

The physiology of the hibernating bear offers a model of translational human research and protection in adverse environments. Space flight, trauma, and intensive care have in common the challenge of preserving organ function in immobility, fasting, and physiologic stress. Immobility is prolonged in hibernating bears, fasting goes on for weeks, and metabolic rate is low with a systemic resilience that would be pathological in the human. This functional redundancy in several body systems offers a framework for clinical strategies that can be transferred to spaceflight, critical care, trauma, and even long-term surgery in the resource-poor environment. The future medicine of extreme environments can be translated from biology that works rather than being established from the ground up.

A key translational possibility would be the induction of synthetic torpor. Earlier rodent studies using adenosine A1 receptor agonists and GABAergic modulation have demonstrated that metabolic rates can be lowered deeply without inducing permanent damage [43]. More recently, researchers have begun to investigate non-pharmacologic induction methods, including ultrasound stimulation of the preoptic area to simulate the initiation of natural torpor [44]. Natural hibernators already display pronounced radio-resistance. Barr and Musacchia reported that hibernating thirteen-lined ground squirrels (*Citellus tridecemlineatus*) survived a 30-day LD50 between 1500 R and 1750 R, whereas active squirrels exhibited an LD50 close to 1100 R, and that survival after high-dose exposure was nearly three-fold longer in torpid than in euthermic animals [45]. Subsequent work shows that torpor up-regulates antioxidant enzymes and suppresses DNA replication, contributing to this protection [46].

Synthetic torpor can reproduce part of this effect. Puspitasari et al. found that rats subjected to a 5′-AMP–induced torpor for six hours immediately after 8 Gy carbon-ion irradiation showed altered survival: one hundred percent of saline-injected rats died between the first eight days, while 92% of 5′-AMP-treated rats died within 11 days, and 8% survived over 30 days [47]. Histology confirmed reduced brain, liver, and lung injury in the torpor group, suggesting combined hypothermia and hypoxia as the protective mechanism.

To date, however, synthetic torpor in non-hibernators has been sustained only in single bouts of six hours [47], and a recent systematic review from 2025 concluded that, although the approach is promising for radiation mitigation, significant knowledge gaps and technical challenges persist, including the absence of data on microgravity-induced musculoskeletal or cardiovascular deconditioning [48]. Thus, while induced torpor offers documented radioprotection and metabolic savings, its efficacy against the collateral effects of weightlessness remains to be demonstrated.

Human torpor protocols will likely be a multilevel, coordinated modulation of multiple systems, including endocrine, circadian, and immune signaling. In humans, it offers two translational avenues: pharmacologic induction, perhaps via adenosine analogs or GABA modulators in ICU or trauma resuscitation; and device-based induction, via targeted ultrasound or neuromodulation to trigger hypometabolism. These could be used in critical illness or for long-term spaceflight to slow metabolism and conserve energy.

As mentioned previously, brown bears switch titin isoforms from N2BA to the stiffer N2B isoform, which maintains myocardial stiffness and prevents chamber enlargement during bradycardia [33]. The role of titin in signaling pathways also highlights the potential of pharmacological targeting of titin spring domains to confer resistance to microgravity or immobilization-induced cardiac atrophy [49]. This would direct the development of drugs mimicking its tension-sensitive behavior to prevent heart failure during protracted unloading, i.e., bedridden patients or space travelers. Mitochondrial adaptations during torpor preserve calcium cycling and prevent arrhythmogenic stimuli even during ischemic or hypothermia states, further supporting the hibernation model for cardiac protection [50]. Medical regimens based on such models would preserve cardiac output independent of physical countermeasures like resistive exercise.

Preservation of skeletal muscle in hibernation is another crucial frontier. Bears inhibit catabolic pathways (Atrogin-1, MuRF1) and enhance oxidation of fatty acids, preserving muscle structure for months without exercise [34]. Low-level muscle micro-movements, possibly neural in origin, may provide adequate mechanical stimulus to preserve contractile proteins [31]. Saxton et al. showed that human myotubes treated with serum from hibernating bears activate Akt/FOXO3a signaling to suppress atrophy genes [35]. These findings hold promise for the development of “hibernation mimetics,” biologic infusions or drugs that can replicate the anti-catabolic signals of hibernation for ICU patients or space travelers under resource-limited conditions [51]. In humans, they may also include recombinant expression of protective factors that mimic the bear’s myokine profile. ICU-acquired weakness and astronaut muscle loss might be prevented by Akt activation, FOXO3a inhibition, or nutrient protocols favoring fatty acid oxidation over proteolysis [35].

Bears also have unmatched renal resistance. Even when they enter prolonged anuria, they do not develop azotemia or electrolyte disturbance. Gut microbiotas facilitate recovery of urea nitrogen through metabolism of retained urea to reabsorbed amino acids [40]. While direct use is limited by human gut microbiome heterogeneity, probiotic modifications or ureolytic bacterial transplantations could be an engineered nitrogen-buffering supplement in patients with reduced renal clearance. It could become increasingly relevant for maintenance of dialysis-free periods during long-duration missions, where logistics make traditional dialysis unfeasible. Microbiome modulation by certain probiotics or diet can help in nitrogen homeostasis in astronauts or with acute renal injury. On the metabolic control side, bears suppress insulin action in the liver, muscle, and fat without causing inflammation or lipotoxicity [37]. This insulin resistance is fully reversible and not similar to pathological diabetes [37]. In human translation, it makes possible the controlled suppression of metabolism either via PPAR modulation or SIRT1 activation to allow for a temporary reduction of metabolic function without organ damage. It is potentially applicable in trauma, multi-organ failure, or suspended animation protocols for interplanetary space flight [52].

The coagulation system itself also changes seasonally. Thienel et al. recognized that pro-coagulant genes F5 and F13A1 are repressed in hibernation, while antithrombotic and endothelium-stabilizing factors PECAM1 and SERPINC1 are preserved [53]. De Vrij et al. corroborated it by showing a reversible, seasonally adapted inhibition of the clotting factors [54]. Translational applications include short-acting anticoagulants or metabolically regulated anticoagulation systems in spacefaring individuals and hospitalized or operated patients. In humans, this would mean that seasonally scheduled dynamic anticoagulation, perhaps by metabolically associated dosing regimens, could reduce immobilization thrombosis without increasing the risk of bleeding on reactivation, especially where fixed-dose anticoagulants are too aggressive or too insensitive.

Radiation is another major hindrance to interplanetary travel. Hibernators, such as brown bears, increase antioxidant capability before and during torpor, raising GPx, SOD, and catalase in various tissues. This pre-torpor defense is anticipatory and begins before torpor, likely in preparation as a buffer for upcoming metabolic stress [31,55]. Clinical applications might involve preconditioning patients and space travelers with antioxidant or cytoprotective medications prior to elective surgery, hypothermic therapy, or sedation.

Cumulatively, these findings explain that the bear’s adaptations are not passive as detailed in Table 1. They are active, future-oriented, reversible processes that protect against physiologic stress. As clinicians and space agencies seek answers to manage long-duration missions, immobilization, or critical illness, nature’s hibernators possess the most advanced model of resilience to be discovered.

## 3. Ophthalmology: Insights into Vision, Optic Nerve, and Retinal Resilience

### 3.1. The Problem: Microgravity-Associated Neuro-Ocular Syndrome (SANS)

Long-duration spaceflight poses vision problems known together as SANS. Flattening of the globe, optic disc edema, choroidal folding, and hyperopic shifts are seen even when measured intracranial pressure falls within normal ranges [24,56]. These changes are assumed to be due to microgravity-induced fluid redistribution, altered cerebrospinal fluid (CSF) dynamics, and venous outflow obstruction, all of which increase the stress over the translaminar pressure gradient [24,56]. Adequately addressing this visual threat requires sensing and activating biologically inspired protective mechanisms.

### 3.2. Hibernator Strategy: Retinal Plasticity and RBM3-Mediated Neuroprotection

Since brown bears have not been the focus of direct ocular physiology research, this review will also refer to other hibernators whose visual capacity has been better elucidated and can offer parallel findings. Most strikingly, perhaps, is the 13-lined ground squirrel, a cone-rich hibernator, which exhibits marked retinal plasticity: optical coherence tomography reveals reversible thinning of the outer retina during torpor and reconstituted structure upon arousal [57,58]. Research indicates that seasonal synaptic remodeling in photoreceptors occurs, potentially implying a model of active structural modification rather than passive decay [59]. These findings reveal that retinal circuits may survive metabolic suppression but retain reversible structural integrity.

At the molecular level, the cold-shock protein RBM3 has been shown to modulate neuroprotective synaptic restoration under hypothermia and hibernation [60,61]. Induction of RBM3 restores synapses in acute stress and neurodegenerative models, and cold-induced RBM3 expression activates downstream effectors like RTN3 to initiate synaptic repair [60,61,62]. These findings establish a mechanistic link between metabolic suppression and neural protection.

**Figure 3:**
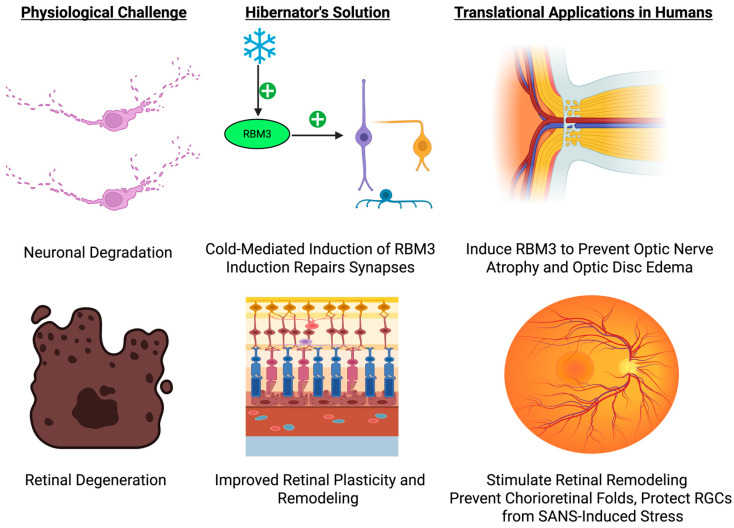
Hibernation-Driven Retinal and Neuro-Ophthalmic Protection: From Mechanism to Translation. This schematic illustrates how hibernation-induced up-regulation of the cold-shock protein RBM3 preserves synaptic integrity and retinal architecture under extreme metabolic suppression, and how analogous strategies such as pharmacologic or device-based RBM3 induction, localized ocular cooling, and adaptive illumination protocols could be deployed to prevent optic nerve atrophy, chorioretinal folding, and vision loss during long-duration spaceflight. Created in BioRender. Shah, J. (2025) https://BioRender.com/2w2lkt1.

While direct ocular physiology research on hibernating brown bears has yet to be done, the observed resilience of retinal structure in hibernating species like the 13-lined ground squirrel provides a strong basis for forming hypotheses. This species has reversible torpor-induced retinal remodeling through photoreceptor and synaptic mechanisms [57,59]. Meanwhile, the cold-shock protein RBM3 has been recognized as a key mediator of neuronal rescue under hypothermia, leading to synaptic reconstitution and cellular survival under conditions of metabolic stress as seen in Figure 3 [60,61,62].

### 3.3. Translational Opportunities: RBM3 Induction, Ocular Preconditioning, and Fluid-Shift Countermeasures

Translating such mechanisms to astronaut vision protection opens several translational avenues. Induction of RBM3 by either pharmacologic agents or gene delivery systems might protect retinal ganglion cells against SANS-induced stress. Directed eye cooling, as from cold-shock RBM3 induction, possibly can precondition retina and optic nerve tissues to be resilient to fluid shift stress. Adaptive illumination environments that simulate cone physiology and synaptic plasticity may decrease visual system stress in space.

In addition to molecular measures, mechanical countermeasures remain essential. Artificial gravity, in the form of centrifuge modules or short-arm rotation, would potentially counteract headward fluid shift and ensure translaminar pressure integrity [63]. In addition, lower-body negative pressure (LBNP) and compressive garments similarly remain potential means for ensuring ocular fluid homeostasis during microgravity simulations [56].

These findings together lay the basis for translational pathways towards preserving astronaut visual integrity, such as localized ocular preconditioning, RBM3-targeted interventions, and adaptive environment design. Future research needs to individually examine ocular resilience in denning brown bears to determine whether they share these neuroprotective adaptations or possess other mechanisms. Such experiments would not only validate extrapolation from other hibernators but also provide direct support for the translational paradigm proposed here for human vision protection in hostile environments.

## 4. Neurological and Neuro-Ophthalmic Preservation

### 4.1. The Problem: Neurovascular Stress in Microgravity and Critical Care

Neurological injury is an important challenge that manifests in both critical care and spaceflight. Symptoms include increased intracranial pressure (ICP), impaired cerebrospinal fluid (CSF) drainage, and SANS as discussed earlier in this paper. These are due to cephalad fluid shifts and glymphatic flow changes in the microgravity environment, which leads to production, clearance, and venous drainage of the CSF being compromised [24]. Similarly, in intensive care settings, neuro-ophthalmic complications can arise secondary to increased ICP, hypoxia, metabolic derangement, systemic inflammation, and all of them ultimately may result in chronic cognitive impairment and ischemic susceptibility.

### 4.2. Hibernator Strategy: RBM3-Driven Neuronal Stability and Glial Quiescence

In contrast, hibernating animals, such as brown bears, and torpid species, such as the thirteen-lined ground squirrel and Djungarian hamster, exhibit neuroprotection when subjected to months of hypoperfusion, immobility, and reduced metabolic rate. A key contributor to this resilience would be the cold-shock protein RBM3 discussed in Section 3 that maintains the integrity of dendritic spines, preserves synapses, and suppresses neuronal apoptosis upon exposure to extreme hypothermia or torpor. In hibernating rodents, RBM3 expression is upregulated during torpor, and it allows direct mediation of recovery from ischemia or traumatic injury [60,64].

**Figure 4:**
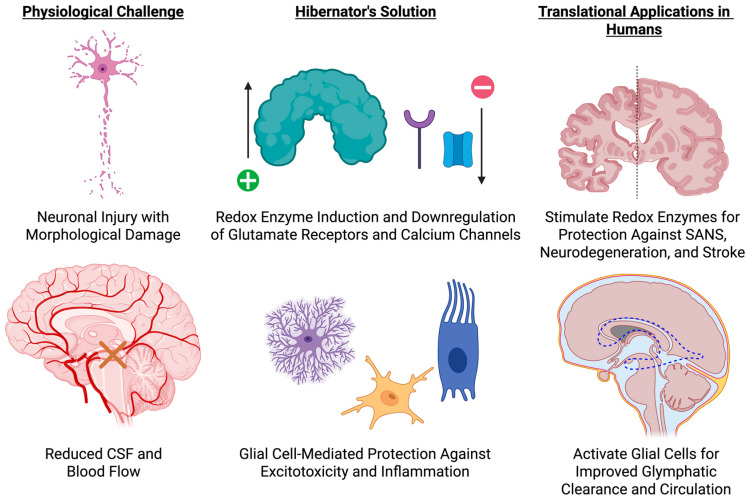
Neuroprotective Adaptations of Hibernators and Translational Strategies. This schematic contrasts the neurological challenges of impaired cerebrospinal fluid (CSF) flow, reduced cerebral perfusion, and excitotoxic injury (**left**) with hibernator-derived protective mechanisms—up-regulation of redox enzymes, down-regulation of glutamate receptors and calcium channels, and glial cell–mediated suppression of inflammation (**center**)—and outlines corresponding human countermeasures, including targeted induction of antioxidant pathways to guard against SANS, stroke, and neurodegeneration, and glial modulation to enhance glymphatic clearance and maintain neural homeostasis. Created in BioRender. Shah, J. (2025) https://BioRender.com/i1pj3pe.

Other than RBM3, glial cells contribute significantly to hibernator neuroprotection. Hibernating hamster and squirrel astrocytes retain perivascular end foot integrity and limit excitotoxic swelling in spite of decreased cerebral blood flow [65]. They show GFAP downregulation, cytoskeletal reorganization, and reduced pro-inflammatory signaling [64,66]. Microglia also exhibit a quiescent morphology, avoiding the synaptic engulfment observed in ischemic or ICU-injured brains [67]. These glial responses are reversible and regulated and, as such, suggest that hibernators actively inhibit neuroinflammation with preservation of brain structure, as shown in Figure 4.

At the level of the neuron, hibernators also show the preservation of dendritic morphology and synaptic density in the face of profound metabolic suppression [65,68]. Cortical and hippocampal neurons preserve cytoskeletal structure and mitochondrial integrity, supported by antioxidant defenses and metabolic remodeling. Some notable transcriptional adaptations would be the downregulation of glutamate receptors and calcium channels to prevent excitotoxicity and the induction of redox enzymes like superoxide dismutase and glutathione peroxidase [65,68]. Arctic ground squirrels withstand >90% reductions in cerebral blood flow during torpor with no ischemic damage, owing to this balance between metabolic suppression and redox regulation [65,69,70].

While intracranial pressure, retinal perfusion, and glymphatic flow cannot be directly measured in hibernating bears and other animals because it is not safe to do so, the absence of SANS-like changes and maintenance of ocular structural integrity of the eye are consistent with the hypothesis of intact neurovascular coupling and autoregulation during hibernation. 13-line ground squirrels retain retinal thickness and do not show evidence of optic nerve sheath distension, which likely reflects a more stable cerebrovascular environment than ICU or microgravity [71].

### 4.3. Translational Opportunities: RBM3 Induction, Astrocyte Modulation, and Glymphatic Support

Translational strategies involve RBM3-mediated therapies to reverse synaptic loss with extended immobility, temperature- or drug-triggered cold-shock protein induction, and metabolic modulators blocking excitotoxic signaling in ischemia or critical illness. Modulation of aquaporin-4 could also restore glymphatic clearance in ICU settings or space travel, and astrocyte-directed treatments can prevent blood-brain barrier disruption and inflammation during disuse states [72]. Additional investigation of brain transcriptomics in bears may identify other evolutionarily conserved pathways for neural resilience.

Hibernators may also offer a systems-level blueprint for the maintenance of brain health in conditions that typically cause degeneration in humans. Glial plasticity, synaptic integrity, metabolic regulation, and inflammation of their hibernators expose new avenues for safeguarding against SANS, ICU delirium, neurodegeneration, and stroke-shifting neuroprotection approaches from reactive to preemptive.

## 5. Cardiovascular Resilience for Extreme Conditions

### 5.1. The Problem: Microgravity and Immobilization-Induced Cardiac Atrophy and Orthostatic Intolerance

The human heart is vulnerable to the synergistic effect of microgravity and immobilization. Fluid shift to the upper body in microgravity reduces venous return to the heart, leading to reduced cardiac preload and approximately a 12% percent reduction of left ventricular mass within days of space travel [73]. This atrophy also causes a profound decrease in stroke volume and orthostatic intolerance upon return to gravity, which places the astronauts at high risk of dizziness and even syncope in resource-poor environments with less access to full rehabilitation systems [74]. On Earth, prolonged bed rest simulates these effects [75]. Investigations show that chronic head-down tilt creates cumulative preload loss, reduced stroke volume, and baroreceptor dysfunction, each reducing cardiovascular tolerance to upright stress [76]. These challenges are rooted not simply in functional dysfunction but in a systemic fragility that undermines mission-critical performance and post-immobilization recovery in the context of austere medical capabilities. Traditional countermeasures such as fluid loading, medical vasoconstrictors, and resistive exercise resist these effects only partially and generally require cumbersome infrastructure, tenuous energy supply, and inordinate crew time, which would be serious limitations in circumstances, such as interplanetary flight or overwhelmed hospitals [77].

### 5.2. The Bear’s Strategy: Myocardial Titin Isoform Shift and RBM3-Linked Cytoprotection

Hibernating mammals, such as brown bears, resist these human limitations in a radically effective manner. Despite weeks or months of extreme bradycardia, with heart rates between 5 and 10 beats per minute, these bears preserve constant ventricular structure, stroke volume, and diastolic function upon arousal [33]. This structural preservation is owed to a changeover in titin isoform expression to the stiffer N2B isoform, which increases myocardial passive stiffness without fibrotic ECM remodeling and preserves effective diastolic filling during reduced preload conditions. In healthy adult human cardiac myocytes, the coexpression of N2B and more compliant N2BA titin isoforms dynamically regulates passive tension, and an isoform shift in their ratio participates in pathological and physiological cardiac adaptations [78,79]. Aside from its mechanical role, titin’s kinase domain transduces stretch into gene regulatory cascades, maintaining myocardial architecture under stress [49]. Cold-shock proteins such as RBM3 have also been described in non-cardiac tissue to be protective against stress-induced cell death through the preservation of mitochondrial integrity, protein translation, and cell viability. It is not a stretch to believe that similar mechanisms, though uncharacterized in the bear heart, are involved in cardiac protection during hypometabolic states [80].

### 5.3. Translational Opportunities: RBM20 Modulation, RBM3 Induction, and Workload Management

**Figure 5:**
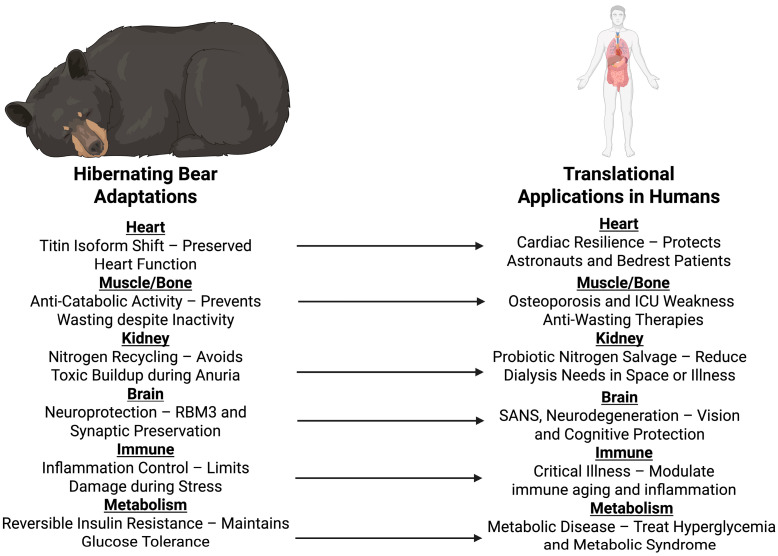
Translational applications of hibernating bears in humans. Physiological mechanisms observed in hibernating bears, like cardiac titin isoform switches, anti-catabolic effects in muscle and bone, nitrogen cycling, neuroprotection, inflammation control, and reversible insulin resistance, are translated into possible human applications. These inform approaches for the preservation of astronauts during long-duration spaceflight, the care of critically ill and immobilized patients, and the engineering of osteoporosis, muscle wasting, kidney disease, neurodegenerative disease, and metabolic disease therapies. Created in BioRender. Shah, J. (2025) https://BioRender.com/h3l3b69.

The remarkable cardiac protection seen in mammals during hibernation offers a rich paradigm for the creation of therapies to preserve cardiac function under conditions of severe stress, as shown in Figure 5. Central to this strategy is the regulation of RBM20 to alter titin isoform expression. In animal models and bioengineered human heart tissues in vitro, knockdown or functional inhibition of RBM20 results in titin isoform composition shifts toward larger and more compliant titin isoforms, which reduces myocardial passive tension and improves diastolic relaxation without pathological remodeling [78,81,82].

Recent work has identified an additional ATP-saving mechanism in bear myocardium that complements the titin-mediated increase in compliance. In left-ventricular strips from hibernating grizzly bears, Van der Pijl et al. (2025) demonstrated a marked increase in the proportion of myosin heads occupying the super-relaxed (SRX) state, a biochemical configuration that hydrolyzes ATP at roughly one-tenth the rate of the disordered-relaxed (DRX) state [83]. Mant-ATP chase assays and X-ray diffraction confirmed that SRX enrichment occurs predominantly in the left ventricle and is accompanied by phosphorylation changes at three residues of the β-myosin heavy-chain coiled-coil region (Ser1300, Thr1309, Ser1543). EvoEF simulations indicated that these modifications stabilize the myosin rod and promote tighter thick-filament packing, while Western blot analyses revealed lower mitochondrial complex II, III, and V abundance in hibernating bears. Together, these alterations reflect a molecular strategy in which cardiac myosin fine-tuning reduces ATP consumption and overall energetic demand during hibernation [83].

Such molecular pathways recapitulate the hibernators’ adaptation to preserve cardiac shape at low preload through modification of their myocardial titin and represents a promising avenue to enhance human diastolic function during states of absent mechanical loading or sustained exercise (Table 2).

Another translational pathway can be realized via the exploitation of RBM3-mediated cytoprotection, which is induced by therapeutic hypothermia and existential stress. RBM3 maintains the mitochondria, prevents apoptosis, and permits protein synthesis during metabolic stress in a range of cell types. While its role in adult myocardium is not studied, RBM3, which that is induced to high levels by hypothermic or ischemic insult in neural and skeletal cells suggests the potential for it to be a systemic cytoprotectant [84,85,86]. A therapy that preconditions the heart through the induction of RBM3 in human myocardium could be utilized to establish underlying metabolic and structural resilience in anticipation of chronic stress or unloading.

A third concept would be selective modulation of atrial workload with preservation of neurohumoral reflexes. Hibernators appear to decrease atrial contribution to cardiac output with neither systemic hemodynamic impairment nor loss of baroreflex sensitivity for cardiovascular homeostasis. Implanted device baroreflex activation therapy (BAT) has been found to demonstrate autonomic modulation and improve cardiac function in patients with heart failure [87,88]. Adaptation of this strategy to transiently reduce atrial workload during periods of immobility can be expected to conserve cardiac energy demands without compromising the reflexive control needed for resumption of upright posture.

A combination of these strategies can generate synergistic therapeutics, such as combining an RBM20-targeted ASO to modulate titin isoforms with RBM3 induction by a mild hypothermic regimen, both administered via wearable or patch-type devices. Such a combination, enhanced by low-level mechanical stimulation like brief-duration passive motion or vibration therapy, may ensure very effective cardioprotection with minimal logistic burden, rendering it highly appropriate for spaceflight or resource-poor clinical environments.

To validate these hypotheses, a stepwise translational roadmap would be taken. Cell and tissue models, such as human iPSC-derived myocardial tissue, would first establish titin compliance and RBM3 expression under simulated inactivity. In vivo testing with animal models, which may include murine hibernation-mimicking protocols, would next determine systemic safety and efficacy. If these are favorable, functional endpoints such as left ventricular diastolic filling and orthostatic tolerance can be investigated in human analog studies in controlled bedrest or tilt-table labs. Operational feasibility and tolerability would be determined in pilot deployment in confined habitat analogues, such as space or submarine analogue facilities.

With this innovation, a transformation in our approach to cardiac preservation would take place, with a shift from the current reactive, resource-intensive support to proactive, biologically inspired mechanisms replicating nature’s most fundamental resilience mechanisms.

### 5.4. Limitations of Current Countermeasures and a Roadmap Forward

Current methods of preserving cardiac function in conditions such as spaceflight and chronic immobilization cannot fully reverse underlying structural deconditioning. Despite rigorous aerobic and resistance exercise training designed to counteract the microgravity state, astronauts nonetheless experience significant cardiac atrophy and reduced orthostatic tolerance after returning to Earth’s gravity [89]. Performance is especially reduced when resources, power, and volume are limited, as observed in the example of long-duration missions aboard the ISS, where exercise equipment on the station routinely failed or operated below design specifications, resulting in a high prevalence of post-flight orthostatic intolerance [90]. Also, a systematic review concluded that passive countermeasures such as vibration, stretch clothing, or passive loading, do not offer any meaningful protection against cardiopulmonary deconditioning or musculoskeletal decline [91].

Pharmacological therapies like midodrine or volume repletion improve hemodynamic symptoms but do not reverse maladaptive myocardial remodeling. These interventions also necessitate continuous supplies, logistic support, and high crew activity that are not feasible in deep-space or resource-limited clinical settings. None of these therapies engage molecular adaptations such as titin splicing modulation or cold-shock protein induction that characterize cardiac resilience in hibernators.

To address this gap, a forward-looking, phased translational roadmap is essential. First, research should utilize in vitro human models, such as iPSC-derived cardiomyocytes, for screening the effects of RBM20 modulation and RBM3 induction on unloading conditions modeled in vitro. Second, animal models should reproduce torpor-like states or prolonged inactivity for assessing systemic safety, cardiac geometry, autonomic responses, and resilience to stress. Thirdly, functional endpoints, such as diastolic filling efficiency, baroreflex sensitivity, and orthostatic performance, must be evaluated via controlled human studies involving analog environments, like head-down tilt or parabolic flights, after molecular or minimal-device interventions. Finally, efficacious protocols must be tested in resource-limited analog habitats, such as submarine bases or Mars analog habitats, for vehicle compatibility, thermal stability, dosing feasibility, and operational viability.

This is a design that extends beyond symptomatic treatment of the reactive type, instead representing a corner-turning into proactive cardiac enablement. It is the strategic insight of hibernator physiology, offering a scalable, low-logistics approach to the maintenance of myocardial integrity in the setting of limited medical resources. By doing so, we move forward toward a model of anticipatory medicine, one based on the lessons of nature’s most supreme survivors.

## 6. Musculoskeletal: Preventing Osteoporosis and Sarcopenia

### 6.1. The Problem: Bone and Muscle Loss in Disuse and Aging

Over the last half-century, international collaboration and technological progress have propelled human space exploration. Ensuring the health of astronauts has become a central concern, and the musculoskeletal system is among the systems most affected by microgravity. On Earth, bone is maintained through a balance between the activity of osteoblasts, which build bone, and osteoclasts, which break down bone. In space, there is an imbalance: osteoclast activity rises while osteoblast activity declines, resulting in increased bone resorption. As a result, astronauts may lose 1–2% of bone density per month in weight-bearing bones such as the femur, hip, and lumbar spine [92].

Muscle atrophy follows a similar trajectory. Without the constant gravitational load, postural and leg muscles lose mechanical stimulation. This reduces protein synthesis and activates pathways that promote protein breakdown [93]. Radiation exposure, poor diet, and limitations of current exercise programs contribute to the problem. Galactic cosmic rays, for example, enhance bone sensitivity to unloading, while poor protein and micronutrient consumption accelerate both bone and muscle loss [94,95,96]. Aerobic and resistance exercise on a daily basis are beneficial, but they do not help enough to completely prevent atrophy or bone loss, mainly due to the loading remaining inadequate and types of training being incompatible with one another [97,98]. Collectively, these adjustments increase the risk of fracture and delay recovery after return to Earth, making potential interplanetary exploration problematic. These musculoskeletal risks also happen in Earth-based settings of disuse: aging, prolonged hospitalization, ICU stays, and neuromuscular disease, all of which mirror the catabolic states induced by microgravity.

### 6.2. The Bear’s Strategy: Structural Preservation Without Load

Hibernating bears avoid disuse osteoporosis despite months of immobility. They do so by suppressing bone resorption while maintaining bone formation, allowing remodeling to continue without a net loss of mass. Gene profiles show reduced osteoclast differentiation and resorption signals, activation of anti-apoptotic pathways, and continuous osteoblast activity. Serum from hibernating bears has been shown to inhibit osteoclast formation and stimulate osteoblast growth in vitro, confirming its protective effect on bone mass [99,100,101].

Their muscles are protected through an additional set of mechanisms. Bears downregulate the ubiquitin-proteasome and autophagy-lysosome systems, reducing protein degradation. Atrophy-related ligases, like MuRF1, are expressed at lower levels, while circulating levels of insulin-like growth factor (IGF) are higher. Total protein content was increased in human muscle cells treated with serum from hibernating bears, most likely through MuRF1 suppression [37].

Complementing these anti-catabolic findings, De Napoli et al. (2025) showed that hibernating brown-bear muscle itself down-regulates ATP turnover to conserve energy [102]. Single-fiber assays revealed an approximate 28% reduction in basal myosin ATPase activity during hibernation relative to the active season [102]. This was linked to diminished phosphorylation of the myosin regulatory light chain, attributable to lower expression of MYLK2, and to a coordinated decrease in oxidative-phosphorylation enzymes identified by quantitative proteomics. Despite the depressed metabolic rate, fiber cross-sectional area, contractile protein composition, and calcium-handling elements remained unchanged, confirming preservation of mechanical competence. Thus, skeletal muscle in hibernating bears functions as an active metabolic regulator, limiting ATP hydrolysis within myofibrils and mitochondria to support whole-body energy economy rather than passively resisting atrophy. By lowering its own ATP demand, skeletal muscle reduces total body energy expenditure and promotes a shift toward lipid utilization with glycogen sparing, thereby contributing to the overall re-organization of whole-body metabolism during hibernation [102].

**Figure 6:**
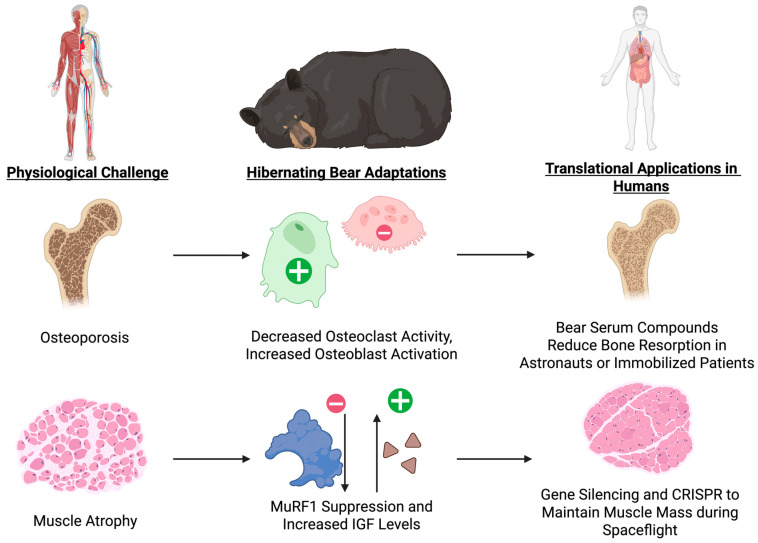
Hibernator-Mediated Preservation of Bone and Muscle and Translational Pathways. This schematic depicts how hibernating bears maintain bone mass by suppressing osteoclast activity while sustaining osteoblast function, and prevent muscle wasting through MuRF1 down-regulation and elevated IGF signaling, and highlights the corresponding human applications such as bear-derived serum factors or mimetics to balance RANKL–OPG signaling and osteoblast activation, and targeted gene or molecular therapies to inhibit atrophy ligases and boost anabolic pathways during disuse or spaceflight.

Further, bears conserve amino acids by maintaining elevated levels of branched-chain amino acids and recycling urea nitrogen. Transcriptomic research shows activation of mammalian target of rapamycin complex 1 (mTORC1) signaling in response to amino acid availability, stimulating protein synthesis while inhibiting autophagy, as well as inhibition of branched-chain amino acid catabolism [38,103]. These findings suggest a well-orchestrated anti-catabolic program capable of protecting both bone and muscle in the absence of mechanical loading (Figure 6).

### 6.3. Translational Opportunities: Modulating Remodeling and Conserving Muscle Mass

Although bear serum is not yet available for clinical application, the findings suggest new therapeutic directions. Current antiresorptive treatments, such as bisphosphonates and denosumab, suppress osteoclast activity but also reduce bone formation indirectly due to the tight coupling of bone remodeling [104]. One option for a translational application might involve finding active compounds or protein fragments in bear serum that regulate RANKL–OPG signaling, osteoblast differentiation, or sclerostin expression. These may be used to design peptide-based therapies or small-molecule mimetics that fine-tune remodeling rather than eliminating it.

Bears, however, are also able to inhibit bone resorption while preserving formation, maintaining healthy turnover [99]. Maintaining this balance could lead to anti-fracture therapies with no long-term side effects seen with present therapies. Models of hibernation also offer additional options for preserving muscle mass, either by targeting mTORC1 activation, modulating IGF pathways, or blocking ubiquitin ligases like MuRF1. New treatment options may include gene silencing of MuRF1 or Atrogin-1 by siRNA, CRISPRa-mediated activation of PGC-1α or IGF-1 in muscle, and pharmacologic mTOR modification using safer nutrient-sensing medications, such as leucine analogs. Beyond pharmacology, genetic and RNA-based approaches may induce temporary reprogramming of the atrophy-related gene expression pathways, and nutrition enhanced with branched-chain amino acids might further facilitate protein conservation.

## 7. Renal and Metabolic: Efficient Fluid and Nitrogen Homeostasis

### 7.1. The Problem: Fluid Redistribution and Catabolic Nitrogen Loss

During spaceflight, astronauts experience changes in their renal hemodynamics due to microgravity-induced cephalad fluid shifts. This leads to a quick decrease in the volume of plasma and extracellular fluid. There is a transient increase in the glomerular filtration rate initially, but renal excretory response to sodium and fluid loads is impaired, most likely owing to increased renal sympathetic activity and stimulation of the renin-angiotensin-aldosterone system [105,106]. These changes are supplemented by intrarenal arterial remodeling due to microgravity, which is defined by elevated renal resistive index, enhanced Rho-kinase-mediated vasoconstriction, and dysfunctional nitric oxide-endothelial nitric oxide synthase-mediated vasodilation, collectively predisposing the kidney to ischemia-reperfusion injury and hindering renal recovery from acute insults [107].

At the tubular and molecular level, cosmic radiation and oxidative stress cause mitochondrial and endothelial dysfunction by generating reactive oxygen species, damaging DNA, and compromising the body’s antioxidant defense systems. Cosmic radiation negatively impacts cellular DNA and causes cells to generate reactive oxygen species, which overwhelm cellular antioxidant enzymes, such as superoxide dismutase, glutathione peroxidase, and catalase. This causes oxidative damage to mitochondrial membranes, proteins, and DNA. This mitochondrial stress reduces ATP synthesis, disrupts cellular metabolism, and promotes cell death and aging in both renal and endothelial cells. All these changes together contribute to reduced natriuresis, elevated blood pressure, altered lipid metabolism, and chronic kidney damage [108,109].

Spaceflight also causes the distal convoluted tubule to increase in size, and the overall tubule density to decrease. It also results in the dephosphorylation of important renal transporters, which may predispose astronauts to nephrolithiasis as a primary renal event rather than a secondary event caused by bone loss. Several factors are responsible for the increased risk of nephrolithiasis in astronauts. Increased excretion of calcium from bone resorption, decreased volume of urine from impaired diuresis, and renal tubular and endothelial dysfunction induced by oxidative stress and radiation exposure are its main causes. Due to these changes, astronauts will need to be closely monitored for renal damage and kidney stones, and precautions must be taken to reduce these risks in long-duration flights [110,111].

### 7.2. The Bear’s Strategy: Renal Suppression Without Toxicity

Bears enter an anuric condition and significantly reduce glomerular filtration while hibernating, but they prevent azotemia by recycling urea nitrogen into protein synthesis, which keeps blood urea nitrogen low and creatinine levels stable. Ornithine and citrulline, two key urea cycle intermediates, accumulate. These changes indicate that nitrogen from the protein degradation is diverted into amino acid and protein synthesis rather than being used to produce urea, thus conserving nitrogen and avoiding its toxic accumulation [38].

In hibernating bears, protective mechanisms are also enhanced, and pro-inflammatory signaling is suppressed. Upregulation of cytokine suppression genes, such as SOCS2, CISH, and SERPINC1, limits renal inflammation and inhibits cytokine-induced damage [112]. Simultaneously, proteins involved in lipid transport, coagulation, and antimicrobial defense are selectively increasing while hydrogen sulfide metabolism is being reprogrammed [113]. By altering hydrogen sulfide metabolism, bears sustain high levels of glutathione, a key antioxidant, in red blood cells. In particular, free cysteine is preserved and bound sulfane sulfur content is reduced, which supports glutathione synthesis and prevents oxidative stress [114].

**Figure 7:**
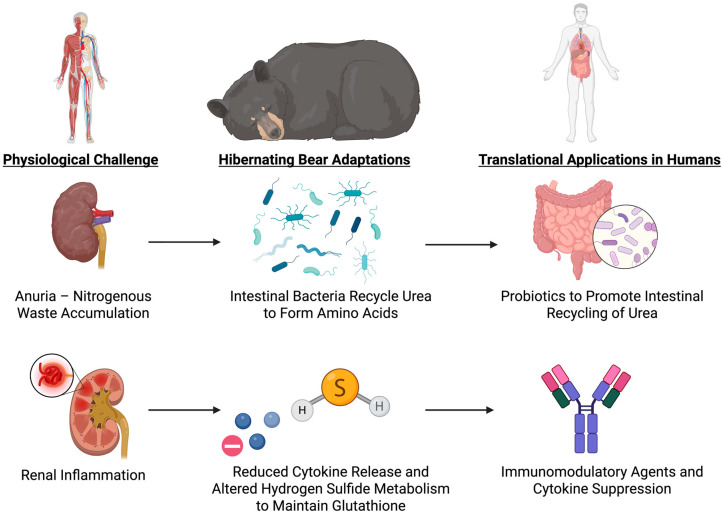
Renal and Metabolic Adaptations in Hibernating Bears and Translational Strategies. This figure illustrates how hibernating bears prevent azotemia by intestinal ureolytic bacteria recycling urea into amino acids and suppressing renal inflammation through cytokine down-regulation and hydrogen sulfide–mediated glutathione preservation, and highlights corresponding human interventions, including probiotic urease supplementation to buffer nitrogen in renal insufficiency and targeted immunomodulatory or gasotransmitter therapies to protect kidney function under stress.

Energy conservation is another critical adaptation. Bears conserve energy during hibernation by increasing the total concentration of plasma proteins through dehydration-induced protein concentration, which avoids energy-costly de novo synthesis. Because bears are months without drinking or urinating, they experience a reduction in plasma volume, concentrating proteins and preserving vital physiological functions, such as immunological defense and coagulation, despite the true levels of the majority of proteins being reduced. Bears can minimize metabolic cost yet sustain essential protein-mediated functions because of this mechanism [113]. Energy conservation also increases through lowered rates of protein breakdown caused by a lower body temperature. Bears reduce their core body temperature by approximately 6 °C during hibernation, which reduces protein catabolism and decreases protease activity. Despite extended durations of inactivity and fasting, this decrease in protein breakdown maintains lean body mass and protects against muscle wasting. Transcriptomic analysis indicating upregulation of protein synthesis and downregulation of genes involved in protein catabolism and autophagy supports the maintenance of muscle and bone integrity [103,115]. Proteins involved in coagulation, lipid transport, and antimicrobial defense, are upregulated during hibernation, while the majority of other proteins are downregulated, because of selective de novo synthesis of key proteins. Bears can sustain tissue protection and essential functions with this specific strategy and at low levels of energy expenditure [38,116]. By maintaining nitrogen balance, avoiding azotemia, facilitating antioxidant defense systems, and selectively facilitating crucial protein synthesis, the molecular mechanisms interact to allow bears to overcome renal challenges, such as anuria and metabolic suppression (Figure 7). This preserves renal and systemic integrity even under prolonged inactivity and metabolic suppression.

### 7.3. Translational Opportunities: Microbial Urease Probiotics, Redox Modulation, and SOCS-Based Immunoprotection

These observations suggest several translational avenues for human medicine. First, the nitrogen-sparing urea recycling mechanism (mediated through microbial urease activity) could be mimicked by administering engineered probiotics to astronauts, especially during periods of synthetic hibernation or other prolonged missions, to buffer nitrogen loads and mitigate any renal insufficiency that might develop. The delivery of urea-degrading bacterial strains could buffer nitrogen loads without dialysis. Second, glutathione preservation through altered hydrogen sulfide metabolism offers the possibility of therapeutic redox balance modulation using sulfur-containing compounds or diet-derived precursors such as N-acetylcysteine. Third, the selective inhibition of protein synthesis, along with preferential preservation of essential coagulation and immune proteins, offers a novel strategy for critical illness involving combined protein preservation and energy restriction. This could be achieved with cell-specific mTOR inhibitors or low-dose nutrient mimetics that suppress non-essential anabolic activity. Lastly, the anti-inflammatory gene expression of hibernators could inform short-term immunomodulation strategies for renal or systemic protection, especially in sepsis or renal ischemia. Agents that induce SOCS2 upregulation or mimic its effects might be beneficial in limiting cytokine-induced organ injury during impaired clearance.

While the clinical literature describes the remarkable molecular and physiological changes that take place in hibernating bears, such as nitrogen-sparing adaptations, enhanced antioxidant defense mechanisms, hydrogen sulfide metabolism remodeling, and selective de novo protein synthesis, no therapeutics currently approved for clinical use take advantage of these mechanisms in humans. However, these findings have generated promising targets. For instance, identification of cytokine suppressor genes (SOCS2, CISH, SERPINC1) suggests potential therapeutic strategies of limiting inflammation and protecting tissues during impaired renal clearance or metabolic stress. Interestingly, studies continue to investigate the genomic and proteomic foundations of hibernating bears with the goal of identifying candidate genes and pathways that can be utilized in human treatment to induce hibernation-like protective states [117,118]. These current activities highlight the promise of developments that could open entirely new avenues for addressing kidney disease, critical illness, prolonged immobilization, and the physiologic challenges of space travel.

## 8. Limitations of the Bear Model

### 8.1. Species Differences

Inherent differences in bear size, metabolism, and evolutionary modifications strictly limit the translational power of bears as model organisms for clinical medicine. Bears have a wide range of body sizes, which are directly influenced by physiological factors, such as the length of hibernation, body temperature and metabolic rate [119]. Large bears, such as the brown and black bears, hibernate shallowly with moderate decreases in body temperature (typically to 30–33 °C) and a deep decrease in the metabolic rate to approximately 25% of basal levels, while maintaining the ability to arouse and respond to external stimuli [119,120,121].

In contrast, small hibernators, exemplified by the Arctic ground squirrel, routinely lower core temperature to 0–5 °C and have been recorded super-cooling to –2.9 °C without tissue freezing, rewarming periodically to normothermia [122,123]. These contrasting strategies reflect both body size and an energetic trade-off: large species rely on ample fat reserves and avoid the high cost of deep re-warming, whereas small species minimize energy expenditure by permitting extreme cooling punctuated by brief arousals. These variations complicate generalization to humans and across species since other species’ metabolic scaling and thermoregulation are fundamentally different from that of bears.

Interspecies differences in metabolic adaptations are also important. Brown bears also exhibit significant seasonal reprogramming of their metabolism, including fat accumulation during hyperphagia, reversible insulin resistance caused by hibernation, and euglycemia conservation during long-term fasting and inactivity. These adjustments are regulated dynamically by adipokines, lipid content, and serum factors, with hibernation characterized by increased lipolysis, increased cortisol, and suppression of mitochondrial respiration and insulin action in muscle and fat [124,125,126,127]. Japanese black bears also have coordinated transcriptional regulation in muscle and adipose tissue: pre-hibernation induction of genes for lipogenesis and the transition to catabolism of fatty acids and protein sparing during hibernation [128]. The novel regulatory mechanisms allowing bears to avoid metabolic syndrome, muscle wasting, and organ damage while fasting for long periods and leading a sedentary lifestyle are not conserved in humans, but they may inform new therapeutic strategies for metabolic disease.

### 8.2. Lack of Ocular and Molecular Detail

The lack of direct information for intraocular pressure (IOP), retinal perfusion, and intracranial pressure (ICP) in hibernating bears imposes very stringent limitations on clinical and research understanding. Without direct measurement, the physiological ranges, the normative levels, and the adaptive mechanisms that are specific to bear hibernation cannot be determined. Such a shortage limits the upper limit of translational information generation to human medicine, particularly for pressure-related pathophysiology, ischemia tolerance, and neuro-ophthalmic adaptation.

Ground squirrel research supports the idea that retinal perfusion is significantly reduced during torpor, but the retina is protected from ischemic injury by radical metabolic and molecular adjustments. Systematic transcriptomic and metabolomic profiling discloses a general suppression of ATP production, a diversion of metabolism from carbohydrates to lipids, and induction of energy-conserving and cellular stress-resistant mitochondrial and DNA packaging pathways under hypoxia [129]. Additionally, cerebral and carotid vascular studies reveal increased vascular resistance and reduced flow during torpor, with adaptive modulating of the contractile pathways (specifically Rho-kinase and nitric oxide signaling) to maintain perfusion and enable recovery upon arousal on a rapid basis [130]. Systemic oxygen saturation falls significantly during torpor and rises rapidly during arousal, in synchrony with rises in heart rate and perfusion [131]. However, these indirect data from ground squirrel hibernation cannot be confidently translated to ocular or intracranial physiology in bears without direct measurement. Without such information, one cannot determine if bears employ similar or disparate protective strategies, limiting translational knowledge for human disease models and pressure- or perfusion-based therapy development.

### 8.3. Logistical and Ethical Constraints

**Figure 8:**
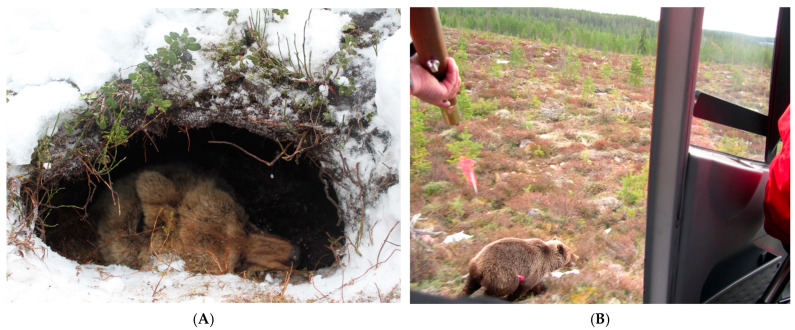
Field study of hibernating brown bears. (**A**) Brown bear in winter hibernation den, demonstrating prolonged intervals of inactivity and fasting without systemic decline. (**B**) Restraint and anesthesia of free-ranging brown bear for physiological monitoring and sampling. Panels (**A**,**B**) together highlight the logistical and ethical barriers discussed in Section 8.3: gaining access to remote dens, the need for anesthesia, and the risk of post-capture stress or den abandonment—all factors that limit sample size and may bias physiological data. Figure reproduced from [9] under Creative Commons Attribution License. (For interpretation of the references to color in this figure legend, the reader is referred to the Web version of this article.)

Animal models are a key component of translational medicine and are employed to translate findings from science into utility in human medicine. One of the fundamental ethical principles of research is balancing the benefits of the research to society against the costs imposed on the animals in research. For hibernating bears, this balance creates a limiting factor: invasive sampling and capturing wild bears for study can profoundly affect their behavior and physiology, with lasting effects that may persist for days to months post-capture. Empirical data reveal that both summer and winter captures, as well as surgical procedures such as muscle biopsies, result in decreased activity and altered heat regulation in brown bears compared to non-captured controls. For example, summer-captured bears exhibit lower movement and reduced body temperatures for at least 14 and 3 days, respectively, with hourly distance reduction by 11%, and winter-captured bears exhibit lowered movement since den emergence until late summer [132]. Figure 8 panel (A) illustrates the process of bear capture, which can be troubling and may lead to den abandonment. Most hibernating bears will leave their dens within days of being brought into captivity, forcing them to find new denning areas and use intermediate resting points, which increases energetic costs and possibly influences their physiological status [133]. Conversely, the behavioral and physiological intrusions present logistical challenges by potentially biasing research results and making it hard to interpret the outcomes under conditions relevant to translational medicine.

Furthermore, there is a risk that biomedical research will divert funds or attention from conservation efforts or inadvertently provide incentives for activities that are detrimental to bear populations. These concerns are exacerbated by the need for repeated sampling or experimental intervention in order to study physiological and genomic adaptation, which can be in conflict with conservation efforts and legal protections of vulnerable bear populations [134]. They include the Andean bear (*Tremarctos ornatus*), or spectacled bear, Asiatic black bear (*Ursus thibetanus*), giant panda (*Ailuropoda melanoleuca*), polar bear (*Ursus maritimus*), sloth bear (*Melursus ursinus*), and sun bear (*Helarctos malanus*) [134]. The fact that these animals are threatened or vulnerable places heavy ethical responsibilities on biomedical research. Endangered and threatened bear populations are usually small, isolated, and genetically distinct and thus highly susceptible to disturbance and subject to further decrease by virtue of research activity. Capture, handling, and invasive procedures cause lasting physiological and behavioral sequelae, stress, and social disruption, which are of concern to animal well-being and potential effect on population viability [135,136,137].

## 9. Conclusions

### 9.1. Summary of Translational Value

Hibernating bears offer a spectacular example of how organisms in nature have adapted to the problem of surviving extreme physiological stress. For several months, these animals remain largely immobile, fasted, and with a dramatically slowed heart rate, yet they recover in springtime without enduring organ failure, bone demineralization, and muscle wasting that humans would develop under similar conditions. Heart studies show their cardiac shape is unaffected despite extreme bradycardia, while muscle and bone are protected by tightly controlled states, preventing tissue decomposition and continuing protein synthesis. Their kidneys conserve nitrogen and electrolytes by recycling waste without the risky accumulation that would be lethal to human health. While their immune and metabolic systems are in homeostasis, they are immune to the chronic inflammation and insulin resistance that typically accompany human disease. Bears are singular in the manner in which these protective mechanisms act synergistically, engaging the cardiovascular, musculoskeletal, renal, immune, and nervous systems as part of an exquisitely coordinated survival mechanism. The survival mechanisms used by bears during hibernation are universally applicable to human health. This bodily resilience explains why the bear is a natural model with deep lessons for the development of new ways of protecting human health from the stresses of space flight, critical illness, and other states of prolonged stress or disuse.

### 9.2. Promise for Future Human Health Applications

The survival strategies bears utilize while hibernating are of universal relevance to human health. During spaceflight conditions, mimicking how they can reduce metabolism and conserve tissues may preclude astronauts from experiencing bone loss, muscle wasting, cardiac remodeling, and eye disease that currently threaten long-duration missions. In critically ill patients in the hospital, these concepts may be applied to them too, where reversible insulin resistance, protective muscle signals, and efficient nitrogen recycling may enable recovery from long-term immobilization. In conditions other than critical illness, these concepts are applicable to the usual pitfalls of aging and offer new therapeutic opportunities for osteoporosis, muscle atrophy, neurodegeneration, and metabolic disease before they irreparably harm. Researchers are already exploring applications in humans, from inducing a brief torpor in human subjects to activating defense proteins like RBM3, to controlling heart proteins that reverse cardiac weakening, and even controlling the microbiome to conserve valuable nutrients. Medicine might shift from reacting to illness to preventing decline entirely by drawing on the inherent resistance of hibernating bears. In so doing, the bear rises above a simple biological fact to become a potential model in the creation of new therapies that could protect human health on Earth and possibly in space as well.

## Figures and Tables

**Table 1:** Translating Hibernator Physiology into Human Countermeasures. Summary of key physiological stressors (cardiovascular compromise, muscle atrophy, endocrine and renal dysfunction, lipid dysregulation), the corresponding adaptive responses observed in hibernating bears, and proposed translational strategies to harness these mechanisms for human protection in settings such as long-duration spaceflight and immobilization.

Physiological Challenges	Hibernating Bear’s Response	Translational Applications in Humans
Cardiovascular Compromise	N2B titin isoform switching in the hibernating bear maintains cardiac elasticity and prevents dilation during prolonged bradycardia. This ensures proper left ventricular filling, and circulation is maintained.	Induction of the stiffer titin isoforms may preserve cardiac dimensions among astronauts while on extended space missions, which would prevent syncope and impaired circulation.
Muscle Atrophy	Hibernating bears can suppress proteolysis and enhance oxidative metabolism to sustain muscle tone and avoid atrophy.	Active compounds from bears can be isolated and tested to have a targeted suppression of proteolysis in humans. Immobilized patients and astronauts may benefit from such treatments to reduce muscle loss.
Endocrine Dysfunction	During hibernation, bears increase insulin resistance to promote breakdown of the body’s stored energy. After hibernation ends, this resistance is reversed.	Having controlled insulin resistance in humans, as opposed to permanent resistance, can allow the body to burn stored fat, thereby treating conditions such as metabolic syndrome. PPAR or SIRT1 modulation may help achieve metabolic modulation.
Renal Dysfunction	Although the kidneys stop producing urine, the hibernating bears have intestinal microorganisms that convert urea into amino acids, ensuring that nitrogenous waste does not accumulate.	Probiotics can be given to astronauts to ensure that they do not develop azotemia due to the buildup of nitrogenous waste, which may occur from renal insufficiency during spaceflight.
Lipid Dysregulation	Increased antioxidant activity and adaptive lipid metabolism prevent vascular damage or atherosclerosis in hibernating bears.	Inflammation and metabolic dysfunction can be prevented in humans by similarly modulating antioxidant activity and lipid metabolism. This may prevent vascular damage in astronauts during spaceflight.

**Table 2:** Cardiovascular Resilience in Hibernating Bears and Translational Countermeasures. This table aligns key cardiac stressors experienced in microgravity or prolonged immobility—orthostatic intolerance, myocardial atrophy and atrial fatigue—with the protective adaptations of hibernating bears, including titin isoform switching to preserve diastolic function, cold-shock protein–mediated cytoprotection of cardiomyocytes and reduced atrial workload, and presents the analogous human interventions such as RBM20 modulation to adjust titin compliance, RBM3 induction through controlled hypothermia and autonomic device-based reduction of atrial force to maintain cardiac performance.

Physiological Challenges.	Hibernating Bear’s Response	Translational Applications in Humans
Orthostatic Intolerance	Hibernating bears preserve their stroke volume, ventricular dimensions, and cardiac preload by expressing the N2B titin isoform. This ensures that the ventricles remain stiff during hibernation, which preserves diastolic function. As a result, syncope does not occur after hibernation, as circulation remains normal.	Atrophy of the left ventricle during spaceflight reduces its filling capacity, which in turn reduces the spaceflight. RBM20 can be silenced to shift human titin isoform composition to more compliant forms, maintaining left ventricular volume and preventing orthostatic intolerance and syncope upon return to Earth.
Loss of Myocardial Architecture	Cold induction of RBM3 offers a cytoprotective effect in hibernating bears. RBM3 enables protein synthesis, prevents cell death, and allows energy use by protecting the mitochondria.	Cold exposure in humans can potentially activate RBM3 to protect against myocardial damage in states of ischemia or bradycardia that may occur in critical care settings or spaceflight.
Atrial Fatigue and Dilation	Hibernating bears reduce the amount of force that the atria produce against a stiffened ventricle to prevent atrial fatigue from occurring.	Atrial workload in humans can be reduced through autonomic modulation with implanted devices that can adjust the heart rate and force of contraction.

## Data Availability

Not applicable.

## Abbreviations

The following abbreviations are used in this manuscript:

ATP	Adenosine Triphosphate
BAT	Baroreflex Activation Therapy
CNS	Central Nervous System
CRISPRa	CRISPR activation
CSF	Cerebrospinal Fluid
ECM	Extracellular Matrix
FOXO	Forkhead Box O
GFAP	Glial Fibrillary Acidic Protein
GPx	Glutathione Peroxidase
GABA	Gamma-Aminobutyric Acid
ICP	Intracranial Pressure
ICU	Intensive Care Unit
IGF	Insulin-like Growth Factor
IOP	Intraocular Pressure
iPSC	Induced Pluripotent Stem Cell
LBNP	Lower Body Negative Pressure
mTOR	Mammalian Target of Rapamycin
mTORC1	Mammalian Target of Rapamycin Complex 1
MuRF1	Muscle Ring Finger Protein 1
NASA	National Aeronautics and Space Administration
PGC-1α	Peroxisome Proliferator-Activated Receptor Gamma Coactivator 1-alpha
PPAR	Peroxisome Proliferator-Activated Receptor
RBM3	RNA Binding Motif Protein 3
RBM20	RNA Binding Motif Protein 20
Rho	Ras Homolog family of GTPases
SANS	Spaceflight-Associated Neuro-ocular Syndrome
SIRT1	Sirtuin 1
SHBG	Sex Hormone-Binding Globulin
SOD	Superoxide Dismutase
SOCS	Suppressor of Cytokine Signaling
SVC	Stroke Volume Compensation
TOR	Target of Rapamycin
